# Changes in the clustering of health-related behaviors during the COVID-19 pandemic: examining predictors using latent transition analysis

**DOI:** 10.1186/s12889-022-13854-x

**Published:** 2022-07-29

**Authors:** Camila Salazar-Fernández, Claire Mawditt, Daniela Palet, Paola A. Haeger, Francisca Román Mella

**Affiliations:** 1 Departamento de Psicología, Universidad de La Frontera, Casilla 54D, 4811230 Temuco, Chile; 2 Departamento de Análisis de Datos, Universidad Autónoma de Chile, Temuco, Chile; 3 NHS England & Improvement, Leeds, UK; 4 Departamento de Ciencias Biomédicas, Facultad de Medicina, Universidad Católica del Norte, Coquimbo, Chile

**Keywords:** Clustering, Alcohol, Smoking, Marijuana, Benzodiazepines, Unhealthy food, Latent transition analysis

## Abstract

The COVID-19 pandemic has had a significant impact on daily life, affecting both physical and mental health. Changes arising from the pandemic may longitudinally impact health-related behaviors (HRB). As different HRBs co-occur, in this study, we explore how six HRBs - alcohol (past-week and binge-drinking), tobacco, marijuana, benzodiazepine use, and unhealthy food consumption - were grouped and changed over time during the COVID-19 pandemic. A sample of 1038 university students and staff (18 to 73 years old) of two universities completed an online psychometrically adequate survey regarding their recalled HRB (T0, pre-COVID-19 pandemic) and the impact of COVID-19 on their behaviors during July (T1) and November (T2). Latent Transition Analysis (LTA) was used to identify HRB cluster membership and how clusters changed across T0, T1, and T2. Four clusters emerged, but remained mainly stable over time: ‘Lower risk’ (65.2–80%), ‘Smokers and drinkers’ (1.5–0.01%), ‘Binge-drinkers and marijuana users’ (27.6–13.9%), and ‘Smokers and binge-drinkers’ (5.6–5.8%). Participants who moved from one cluster to another lowered their HRB across time, migrating from the ‘Binge-drinkers and marijuana users’ cluster to ‘Lower risk’. Participants in this cluster were characterized as less affected economically by the COVID-19 pandemic, with lower reported stress levels, anxiety, depression, and loneliness than the other clusters. Our results provide evidence of how HRBs clustered together and transitioned longitudinally during the COVID-19 pandemic. HRB clustering across time offers a valuable piece of information for the tailoring of interventions to improve HRB.

**Supplementary Information:**

The online version contains supplementary material available at 10.1186/s12889-022-13854-x.

## Introduction

Alcohol, tobacco, marijuana, benzodiazepine use, and unhealthy food consumption are among the major causes of morbidity and mortality worldwide [1]. Because these behaviors contribute to the development of non-communicable diseases such as cardiovascular disease, cancer and mental disorders, they are considered health-related behaviors (HRBs). HRBs do not occur in isolation but rather commonly co-occur or cluster together [2–5]. HRBs are more detrimental to health and wellbeing in clusters than individually; moreover, they have a higher combined risk of early mortality [6, 7]. Understanding how HRBs cluster together may provide relevant information for effective interventions aimed at the higher risk groups [8, 9]. Accordingly, the WHO has recommended tackling chronic diseases based on a preventive approach to multiple HRBs [10].

Literature on clustering HRBs has shown that alcohol and tobacco consumption are usually grouped together [3, 4, 11]. Furthermore, research has also found co-occurrence patterns among alcohol, marijuana, and tobacco consumption [12–14] and between alcohol, tobacco, and poor diet [15, 16]. However, to our knowledge, no study has reported how alcohol, tobacco, marijuana, benzodiazepine use, and unhealthy diet cluster together and how the clustering of HRBs could be affected and change over time, especially during the COVID-19 pandemic.

Engaging in multiple HRBs can be affected by external events, such as economic, traumatic, natural, and environmental crises [17–20]. Evidence reporting the effects of the COVID-19 pandemic has shown an increase in negative emotional states such as anxiety, depression, stress, confusion, anger, and frustration, which are associated with being involved in multiple HRBs [21–28]. The restriction of social and recreational contexts due to measures implemented to prevent the spread of COVID (i.e., lockdowns, work from home, and social distancing) raises the need to monitor how HRB co-occurrence has changed throughout the pandemic.

During a crisis, such as the pandemic, two scenarios are expected: increased HRB due to high levels of emotional distress [29] or decreased HRB due to reduced accessibility to substances [18, 30]. Current evidence on changes in individual HRBs during the pandemic has reported support for both possible results: an increase and decrease in alcohol [31–33] and tobacco consumption [34–36]. Meanwhile, marijuana use has remained stable [37] or has increased [38–40], especially in people who have been in lockdown [41]. In relation to unhealthy food consumption, results finding an increase are robust [21, 40, 42–44]. Regarding the use of benzodiazepines, a few studies have reported a reduction compared to previous years [45]; however, data from a non-probabilistic and self-selected sample survey in Chile have shown an increase in the use of benzodiazepines without prescription when compared with pre-pandemic data [46]. Benzodiazepine is a psychotropic drug with sedative/anxiolytic and hypnotic effects which acts by enhancing the effects of gamma-aminobutyric acid (GABA), an inhibitory neurotransmitter in the central nervous system [47].

While previous studies have shown changes in substance use and unhealthy food consumption during the pandemic, to our knowledge, there is no evidence on how these HRBs co-occur and whether this clustering has changed during the pandemic. The present study focuses on HRB during the COVID-19 pandemic in a university setting (students and staff). Using latent transition analysis, we explored how HRBs clustered during the pandemic and whether participants shifted to different clusters according to their changes in behavior during the pandemic period. Finally, we also examined emotional distress and social demographic participant characteristics that shifted from one cluster to another across different times during the pandemic.

## Material and methods

### Participants and procedure

A purposive non-probabilistic longitudinal survey was implemented in July (Time 1, T1) and November (T2), 2020 to collect data about HRB among university students and staff (academic and non-academic) from two universities, one located in the southern and one in the northern region of Chile. In the first assessment, participants retrospectively reported their HRB prior to the COVID-19 pandemic (T0, *n* = 1038) and during the pandemic (T1, *n* = 1038 and T2, *n* = 430). Informed consent to participate in the study was obtained from all participants prior to complete online surveys (about 15 minutes). The Ethics Committee of both universities approved this study (Res. 086/20 on July 2020 and 11/2020 on June 2020). Sample characteristics are described elsewhere in Salazar-Fernández et al. [22] and Salazar-Fernández et al. (2021b).

### Measures

Six HRBs were measured for cluster membership: cigarettes per day, past-week alcohol and unhealthy food consumption, monthly frequency of binge drinking, marijuana and benzodiazepine use. See Table 1 for the items, response options and treatment for the HRB variables used to examine clustering.

**Table 1:** HRB variables used for clustering

Variables/Covariables		Item	Response option	Variable treatment
HRB for clustering	Cigarettes	How many cigarettes have you smoked per day?	From 0 to 40 cigarettes or more.	-
	Alcohol consumption	In the past week, how many drinks did you consume?	From 0 to 20 drinks or more.	Weekly quantity of drinks was calculated based on the number of drinks consumed each day in the previous week.
	Unhealthy food consumption	During the last week, on how many days have you consumed fried meals, sugary drinks, desserts or candies, unhealthy snacks and fast food.	From 0 to 7 days.	Responses were summed to provide a total score indicating total score of weekly unhealthy food consumption.
	Binge drinking	Thinking back to the past 30 days, how often have you had 5 or more drinks on a single occasion?	Responses were scored on a 6-point scale: never, once, twice, 3 to 5 times, 6 to 9 times, and 10 or more times.	-
	Marijuana use	Have you smoked marijuana?	Yes/No	-
	Benzodiazepines use	Have you taken self-medicated tranquilizers?	Yes/No	-

Several variables were used as covariates to analyze their association with the resulting clusters. These variables were: age, gender, ethnic minority group (participants could identify as belonging to an ethnic group if they were from one of the ten ethnic groups recognized by the law of Chile: Atacameño or Likán Antai, Aymara, Coya, Diaguita, Kawésqar, Mapuche, Quechua, Rapa Nui, and Yagán or Yámanam or not), and region (if they live in the northern, southern or another region of Chile). Emotional distress was measured using the short version of the Depression, Anxiety, and Stress Scale, DASS-21 [48, 49] using a 4-point severity scale. The perceived impact of COVID-19 was assessed through three questions inquiring about its interpersonal, health and economic effects (5-point scale from “not at all” to “a lot”). Finally, loneliness was assessed using a frequency item (4-point scale from “never” to “always or almost always”).

### Statistical analyses

#### Latent transition analysis

Latent Transition Analysis (LTA) was used to identify HRB cluster membership across the three data times (i.e., T0, T1 and T2). According to Collins and Lanza [50] LTA models consist of three parameters: (a) the probability of being in a particular HRB cluster at each time; (b) the probability of the person’s response to each HRB variable given their HRB cluster membership at each time; (c) the probability of transitioning to a different HRB cluster at a subsequent time (i.e., T1 and T2), given their HRB cluster membership at baseline (i.e., T0). To determine an optimal number of clusters, several LTA models were estimated, adding another cluster (*k*) to each consecutive model and comparing entropy and fit indices to the previous model (*k*-1). Entropy above 0.8 indicates low classification error in HRB cluster assignment [51]. Fit statistics included log-likelihood, Akaike Information Criterion (AIC), Bayesian Information Criterion (BIC), and adjusted Bayesian Information Criterion (aBIC), whereby a lower number indicates an improvement in model fit [50, 52]. Missing data was handled in the LTA models using a Full Information Maximum Likelihood Function, under the missing at random (MAR) assumption [53], using Mplus version 8.1 [54].

#### Cluster membership and covariates

Bivariate analyses, using one-way ANOVA, Kruskal-Wallis and/or Chi-square Fishers Exact test, were undertaken to show the means and/or proportions for covariates according to cluster membership. The effect size for continuous variables was addressed using eta squared. Following the bivariate analyses, multinomial logistic regression models were run in Stata/SE version 15 [55]. ,These models tested whether associations between HRB cluster membership and covariates identified through the bivariate analyses remained statistically significant after mutually adjusting for other covariates in the model. To prevent under-estimated standard errors in the regression models, we adjusted for classification error associated with cluster assignment [56].

## Results

Our sample age was 18 to 73 years (M = 29.52, SD = 11.66), 69% were female, and only 18.4% declared themselves as belonging to an ethnic minority group. Most of these were Mapuche (16.2%) and Aymara (1.6%), the rest were Diaguita, Kawésqar, and Quechua. Of the total sample, 67.3% lived in the southern region. Several descriptive statistics and frequency analysis were performed on the total sample to explore the health-related variables (see Table 2). Then, we proceed with the LTA.

**Table 2:** Health risk behaviors among the university sample (*n* = 1038)

	Total sample	Cluster 1 ‘Lower risk’	Cluster 2 ‘Smokers and drinkers’	Cluster 3 ‘Binge-drinkers and marijuana users’	Cluster 4 ‘Smokers and binge-drinkers’
**Cluster prevalence**	**n**	**n (%)**	**n (%)**	**n (%)**	**n (%)**
T0	1038	677 (65.2)	16 (1.5)	287 (27.6)	58 (5.6)
T1	1038	814 (78.4)	13 (1.3)	157 (15.1)	53 (5.1)
T2	430	830 (80.0)	4 (0.01)	144 (13.9)	60 (5.8)
**Mean and standard errors (S.E)**	**Mean (S.E)**	**Mean (S.E)**	**Mean (S.E)**	**Mean (S.E)**	**Mean (S.E)**
Cigarettes smoked per day	0.56 (1.76)	0.04 (0.01)	10.60 (0.37)	0.28 (0.05)	4.44 (0.13)
Total weekly number of alcoholic drinks	2.55 (3.49)	1.12 (0.08)	6.43 (1.52)	5.36 (0.70)	4.46 (0.54)
Total weekly score of unhealthy food consumption	8.89 (5.87)	8.82 (0.19)	8.92 (1.33)	10.04 (0.41)	9.87 (0.74)
**Item response probabilities (IRP) and standard errors (S.E)**	**(%)**	**IRP (S.E)**	**IRP (S.E)**	**IRP (S.E)**	**IRP (S.E)**
Monthly frequency of binge drinking					
*Never*	52.5	0.84 (0.03)	0.50 (0.09)	0.08 (0.03)	0.39 (0.05)
*Once*	27.6	0.14 (0.02)	0.12 (0.05)	0.39 (0.05)	0.23 (0.04)
*Twice*	11.4	< 0.01(< 0.01)	0	0.30 (0.03)	0.16 (0.03)
*3 to 5 times*	6.4	< 0.01(< 0.01)	0.24 (0.13)	0.20 (0.04)	0.17 (0.04)
*6 to 9 times*	1.3	0	0.15 (0.11)	0.02 (0.01)	0.05 (0.02)
*10 times or more*	0.3	0	0	0.01 (0.01)	0
Marijuana use					
*No*	81.6	0.88 (0.02)	0.65 (0.10)	0.44 (0.05)	0.62 (0.05)
*Yes*	17.9	0.12 (0.02)	0.35 (0.10)	0.56 (0.05)	0.38 (0.05)
Benzodiazepine use					
*No*	86.3	0.90 (0.01)	0.79 (0.07)	0.80 (0.03)	0.80 (0.04)
*Yes*	13.2	0.10 (0.01)	0.21 (0.07)	0.20 (0.03)	0.21 (0.04)

### Latent transition analysis

Based on the LTA model fit statistics (see Supplementary Table 1), the 4-cluster model was selected. The 2- and 3- cluster models had a higher log-likelihood, AIC, BIC and aBIC statistics than the 4-cluster model. The 5-cluster model could not be estimated, likely due to data sparseness. The HRB cluster assignment classification error in the original latent variable was considered to be low for all of the models, indicated by an entropy of 0.9.

### Cluster patterns and membership

Based on the LTA item response probabilities and means (see Table 2), the 4 clusters were labelled as follows: ‘Lower risk, ‘Smokers and drinkers’, ‘Marijuana and alcohol users’ and ‘Smokers and binge-drinkers’. The ‘Lower risk’ cluster was the largest and had low-risk levels of HRB in comparison with the other clusters. Very few members of this cluster smoked cigarettes, engaged in binge drinking or substance use. The ‘Smokers and drinkers’ cluster was very small (0.01 to 1.5%), with riskier HRB. Members of this cluster smoked more cigarettes per day, consumed more alcoholic drinks in the previous week and reported a higher frequency of binge drinking than the other clusters. Members of the ‘Binge-drinkers and marijuana users’ cluster, the second largest, had very few daily smokers but a higher proportion smoked marijuana relative to the other clusters. The consumption of unhealthy food was also higher in this cluster than the others. The ‘Smokers and binge-drinkers’ cluster was also very small (5.1 to 5.8%). Members of this cluster smoked fewer cigarettes per day, reported a higher frequency of binge drinking, and had a higher consumption of unhealthy food than members of the ‘Smokers and drinkers’ cluster.

The LTA transition probabilities (see Table 3) indicated that most participants remained in the same HRB cluster across the three times. However, amongst those who did move, 43% of the ‘Binge-drinkers and marijuana users’ cluster transitioned to the ‘Lower risk’ cluster at T1 and 16% did so at T2. Further analyses showed that those who moved from the ‘Binge-drinkers and marijuana users’ cluster were more likely to be younger (≤ 25 years old) than those who stayed (see Supplementary Table 2).

**Table 3:** Transition probabilities of health risk behaviors clusters

	Cluster 1	Cluster 2	Cluster 3	Cluster 4
**Transition probabilities from T0 (rows) to T1 (columns)**				
Cluster 1: ‘Lower risk’	**0.99**	0^a^	0^a^	0.01
Cluster 2: ‘Smokers and drinkers’	0.37	**0.31**	0.13	0.19
Cluster 3: ‘Binge-drinkers and marijuana users’	0.43	0^b^	**0.52**	0.05
Cluster 4: ‘Smokers and binge-drinkers’	0.33	0.14	0.06	**0.47**
**Transition probabilities from T1 (rows) to T2 (columns)**				
Cluster 1: ‘Lower risk’	**0.98**	0^a^	0.02	0.01
Cluster 2: ‘Smokers and drinkers’	0^a^	**0.38**	0^a^	0.63
Cluster 3: ‘Binge-drinkers and marijuana users’	0.16	0^a^	**0.82**	0.03
Cluster 4: ‘Smokers and binge-drinkers’	0.27	0.05	0.11	**0.58**

Transition probabilities in bold correspond to staying in the same HRB cluster

Transition probabilities sum to 1.0 (with rounding error) across rows

^a^Transitions not estimated in model but instead fixed to 0 in Mplus

^b^Transitions < 0.01 and rounded to 0

### Cluster membership and covariates

Bivariate analyses uncovered associations between HRB cluster membership and covariates measured at the same time (see Table 4, Supplementary Tables 3 and 4). HRB cluster membership and stress and anxiety scores were significant (*p* < 0.05, both *η*^2^ = .010) at T1 and T2. Members of the ‘Lower risk’ cluster tended to have lower levels of stress and anxiety, than the other three clusters. Age was associated with HRB cluster membership at T0 and T1, but not T2. Members of the ‘Smokers and drinkers’ cluster tended to be older than the ‘Lower risk’ cluster, and those in the ‘Binge-drinkers and marijuana users’ cluster were younger than the ‘Lower risk’ cluster at T0. However, due to movement of younger participants from the ‘Binge-drinkers and marijuana users’ cluster to the ‘Lower risk’ cluster (as mentioned above), the age of members of both clusters was similar at T1. Feeling lonely was associated with HRB cluster membership at T0, but not at T1 or T2, with members of the ‘Lower risk’ cluster feeling less lonely than those in the other clusters. The perceived negative economic impact of the pandemic was significant at T1, members of the ‘Lower risk’ cluster reported being less affected in economic terms than members of the other clusters. None of the remaining covariates showed associations with the HRB clusters.

**Table 4:** Bivariate analyses using health risk behaviors cluster membership and covariates at T1

	Cluster 1 ‘Lower risk’ ***n*** = 814 (79)	Cluster 2 ‘Smokers and drinkers’ ***n*** = 14 (1)	Cluster 3 ‘Binge-drinkers and marijuana users’ ***n*** = 157 (15)	Cluster 4 ‘Smokers and binge-drinkers’ ***n*** = 52 (5)
	n (%)	n (%)	n (%)	n (%)
Age^**^				
*< =25*	459 (80)	3 (1)	84 (15)	26 (4)
*26+*	355 (76)	11 (2)	73 (16)	26 (6)
**Gender**				
*Female*	566 (79)	9 (1)	110 (15)	31 (4)
*Male*	235 (77)	4 (1)	46 (15)	22 (7)
**Loneliness**				
*Never/Rarely/Sometimes*	487 (80)	8 (1)	83 (14)	34 (5)
*Frequently/Always*	326 (77)	6 (1)	74 (17)	19 (4)
Perceived negative impact on economic or employment status^**^				
*Not at all/A little/Some/*	654 (80)	12 (1)	119 (15)	35 (4)
*Quite a bit/A lot*	159 (73)	2 (1)	38 (18)	18 (8)
**Perceived negative impact on personal relationships with family or friends**				
*Not at all/A little/Some/*	586 (80)	9 (1)	101 (14)	35 (5)
*Quite a bit/A lot*	227 (74)	5 (2)	56 (18)	18 (6)
**Perceived negative impact on own or loved ones’ health**				
*Not at all/A little/Some/*	665 (80)	10 (1)	121 (14)	39 (5)
*Quite a bit/A lot*	148 (73)	4 (2)	36 (18)	14 (7)
**Ethnic minority group**				
*No*	667 (79)	14 (2)	122 (14)	44 (5)
*Yes*	147 (77)	0	35 (18)	9 (5)
**Region**				
*Northern region*	170 (78)	2 (1)	37 (17)	10 (5)
*Southern region*	532 (79)	10 (1)	105 (16)	30 (4)
*Another region*	85 (77)	2 (2)	12 (11)	12 (11)
**Depression** *Mean (SD)*	7.00 (5.54)	6.75 (5.21)	8.13 (5.31)	8.56 (6.81)
**Anxiety** Mean (SD)^*±^	5.04 (5.20)	6.83 (8.02)	6.08 (5.15)	6.76 (6.11)
**Stress** Mean (SD)^*±^	7.86 (5.44)	7.33 (7.57)	9.26 (4.86)	9.16 (6.61)

^*^p < 0.05 using one-way ANOVA

^*±*^*p* < 0.01 using Kruskal-Wallis

^**^p < 0.01 using Fisher’s Exact test

Multinomial logistic regression models, adjusting for classification error and other covariates in the model (see Supplementary Tables 5, 6 and 7), identified that members of the ‘Binge-drinkers and marijuana users’ cluster were younger than those in the ‘Lower risk’ cluster at T0 and older at T1. Members of the ‘Binge-drinkers and marijuana users’ cluster had a greater risk of feeling lonely than those in ‘Lower risk’ cluster at T0. The perceived negative economic impact of the pandemic remained significant at T1, members of the ‘Smokers and binge-drinkers’ cluster were more negatively affected than members of the ‘Lower risk’ cluster. Moreover, at T1, members of ‘Smokers and drinkers’ cluster had higher levels of anxiety than the ‘Lower risk’ cluster.

## Discussion

In this study, we explored HRB clusters and changes during the COVID-19 pandemic. At T0, we identified four clusters ‘Lower risk’, ‘Smokers and drinkers,’ ‘Binge-drinkers and marijuana users,’ and ‘Smokers and binge-drinkers.’ The LTA showed that most participants remained in the same HRB cluster during T1 and T2, but those who moved tended to improve their HRB across time, migrating to the ‘Lower risk’ cluster.

Our ‘Lower risk’ cluster is consistent with previous pre-pandemic studies that have found a predominant ‘healthier’ cluster grouping of most participants [3]. We also found that members of the ‘Lower risk’ cluster were older, were less affected economically by the COVID-19 [17], and reported lower levels of stress, anxiety, depression, and loneliness than the others clusters [57]. Furthermore, members of ‘Binge-drinkers and marijuana users,’ the second-largest cluster found in our study, were younger and reported a greater risk of loneliness than the other clusters [58–60].

We found that young people (≤ 25 years old) were more likely to move from the ‘Binge-drinkers and marijuana users’ cluster to the ‘Lower risk’. This finding is consistent with other study with university students were almost 25% of participants reported a decrease in binge drinking, however reported no change in marijuana use [37]. This could be because of the social nature of consumption among young people, which has been particularly affected by the measures to contain the COVID pandemic [32, 37]. Thus, alcohol use prevention policies at the university level must not only encourage a reduction in social and commercial access to alcohol and tobacco [61], but also promote alternative substance-free social/recreational activities [62].

In contrast to other results [63, 64], in this study, we found that weekly unhealthy food consumption was not a distinctive HRB for clustering since it was high in all four clusters. These findings are consistent with other studies on the Chilean population before [65] and during the COVID-19 pandemic reporting an increase in unhealthy food consumption [66, 67] and excess weight [68].

Our findings confirm previous evidence that multiple HRBs cluster together [2, 37], particularly regarding the strong association between smoking and alcohol consumption or heavy episodic drinking [2–4]. Hence, person-centred strategies should be considered when targeting any HRB. Our research provides useful information identifying HRB clusters and how they change longitudinally during the COVID-19 pandemic. Our results suggest interventions targeting depression, anxiety, stress, and loneliness are needed to facilitate improvements in HRB regarding cluster membership. Therefore, academic institutions should focus on strengthening mental health prevention and promotion for students and staff [60, 69]. Institutions also need to improve health education to prevent HRB [70], especially unhealthy food consumption, which was generally observed in the sample, and can lead to greater health problems [71].

Despite its strengths, our study had some limitations: (1) the data relies on self-reported measures of HRB, which can be biased (i.e., social desirability); this could explain the larger size of the ‘Lower risk’ cluster compared to the smaller sizes of ‘Smokers and drinkers’ and ‘Smokers and binge-drinkers’ [72]; (2) the online collected sample is limited to students and university staff (mostly women) who have not been directly exposed to the economic effects of the pandemic (e.g., maintained their jobs and have been teleworking); (3) as this study used self-selection sampling, the results might be affected by selection bias; however, given the pandemic restrictions, it would not have been feasible to conduct a more robust sampling; (4) we only explored HRB after 4 months of the pandemic, thus it is possible that larger lags could have implied more transitions of participants between clusters as opposed to participants remaining in the same clusters, and (5) as in many studies [73, 74], the measures for marijuana and benzodiazepines use in this study were categorical and not continuous, as Becher [75] has stated this could lead to model misspecification and high residual confounding. Therefore, future studies should consider addressing these limitations and the possible increase in HRB associated with lifting the COVID-19 restrictions because social and recreational gatherings will eventually return to daily life dynamics, especially among university students.

## Conclusions

During COVID-19, we identified 4 clusters of health-related behaviors in adults and how they transitioned over time between clusters. Most of the participants remained stable during follow up and those who moved were more likely to improve their HRB transitioning to the ‘Lower risk’ cluster. This was especially true in young people who changed from ‘Binge-drinkers and marijuana users’ cluster to the ‘Lower risk’ cluster. Without the social restrictions due to the pandemic, substance-free social opportunities should be provided for young people. Our results show that HRBs tend to cluster, so any prevention effort should take into consideration the ways in which people become involved in different HRBs and the factors leading to these behaviors.

## Supplementary Information

**Additional file 1: Supplementary Table 1.** Fit indices for the latent transition analysis. **Supplementary Table 2.** Covariates of those who stayed in ‘Binge-drinkers and marijuana users’ cluster and those who moved to *‘Lower risk’* cluster between T0 and T1. **Supplementary Table 3.** Bivariate analyses HRB cluster membership and covariates at T0. **Supplementary Table 4.** Bivariate analyses HRB cluster membership and covariates at T2. **Supplementary Table 5.** Multinomial logistic regression model estimating association between cluster membership at T0 and covariates, adjusting for classification error. **Supplementary Table 6.** Multinomial logistic regression model estimating association between cluster membership at T1 and covariates, adjusting for classification error. **Supplementary Table 7.** Multinomial logistic regression model estimating association between cluster membership at T2 and covariates, adjusting for classification error.

## Data Availability

The datasets used and analysed during the current study are available from the corresponding author on reasonable request.
