# Supplementary material for: Changes in the clustering of health-related behaviors during the COVID-19 pandemic: examining predictors using latent transition analysis
Source: BMC Public Health. 2022 Jul 29;22:1446. doi: 10.1186/s12889-022-13854-x (PMC9338510; doi:10.1186/s12889-022-13854-x)
Supplement: Supplementary file 1 — Additional file 1: Supplementary Table 1. Fit indices for the latent transition analysis. Supplementary Table 2. Covariates of those who stayed in ‘Binge-drinkers and marijuana users’ cluster and those who moved to ‘Lower risk’ cluster between T0 and T1. Supplementary Table 3. Bivariate analyses HRB cluster membership and covariates at T0. Supplementary Table 4. Bivariate analyses HRB cluster membership and covariates at T2. Supplementary Table 5. Multinomial logistic regression model estimating association between cluster membership at T0 and covariates, adjusting for classification error. Supplementary Table 6. Multinomial logistic regression model estimating association between cluster membership at T1 and covariates, adjusting for classification error. Supplementary Table 7. Multinomial logistic regression model estimating association between cluster membership at T2 and covariates, adjusting for classification error. [file 12889_2022_13854_MOESM1_ESM.docx]

Supplementary Files

**Supplementary Table 1.** Fit indices for the latent transition analysis.

| **Fixed^a^** | **Log-likelihood** | **AIC** | **BIC** | **aBIC** | **Entropy** |
| --- | --- | --- | --- | --- | --- |
| 2 cluster | -23158.443 | 46392.885 | 46580.797 | 46460.104 | 0.979 |
| 3 cluster | -22224.836 | 44555.671 | 44817.759 | 44649.424 | 0.979 |
| **4 cluster** | **-21626.717** | **43405.435** | **43781.258** | **43539.873** | **0.902** |
| 5 cluster^b^ | -21214.311 | 42634.621 | 43143.962 | 42816.820 | 0.930 |

*Note.* Preferred model highlighted in bold

^a^ Measurement invariance assumed over time

^b^ The 5-cluster model was unidentified, the log-likelihood did not replicate. Fit statistics may not be reliable, they are included for comparative purposes.

**Supplementary Table 2.** Covariates of those who stayed in ‘Binge-drinkers and marijuana users’ cluster and those who moved to *‘Lower risk’* cluster between T0 and T1.

|  | **T0 to T1** | |
| --- | --- | --- |
|  | **Stayed in ‘Binge-drinkers and marijuana users’ cluster (*n* = 152, 56%)** | **Moved to ‘Lower risk’ cluster**  **(*n* = 120, 44%)** |
|  | **n (%)** | **n (%)** |
| **Age***^**^*  *<=25*  *26+* | 81 (48)  71 (70) | 89 (52)  31 (30) |
| **Gender**  *Female*  *Male* | 108 (56)  44 (57) | 85 (44)  33 (43) |
| **Loneliness**  *Never/Rarely/Sometimes*  *Frequently/Always* | 110 (56)  42 (57) | 88 (44)  32 (43) |
| **Perceived negative impact on economic or employment status**  *Not at all/A little/Some/*  *Quite a bit/A lot* | 115 (56)  37 (57) | 92 (44)  28 (43) |
| **Perceived negative impact on personal, family or friends relationships**  *Not at all/A little/Some/*  *Quite a bit/A lot* | 98 (54)  54 (59) | 83 (46)  37 (41) |
| **Perceived negative impact on own or loved ones health**  *Not at all/A little/Some/*  *Quite a bit/A lot* | 117 (57)  35 (53) | 89 (43)  31 (47) |
| **Ethnicity**  *No*  *Yes* | 117 (55)  35 (60) | 97 (45)  23 (40) |
| **Region**  *Region of Coquimbo*  *Region of La Araucanía*  *Another region* | 37 (65)  100 (56)  12 (43) | 20 (35)  79 (44)  16 (57) |
| **Depression** *Mean(SD)* | 8.19 (5.37) | 8.47 (5.49) |
| **Anxiety** *Mean(SD)* | 6.07 (5.19) | 6.66 (5.80) |
| **Stress** *Mean(SD)* | 9.21 (4.85) | 9.57 (5.25) |

## *Note.* ^**^ *p* *≤* 0.05 using Fisher’s Exact test.

**Supplementary Table 3.** Bivariate analyses HRB cluster membership and covariates at T0.

|  | **Cluster 1**  **‘Lower risk’**  **(n=677)** | **Cluster 2**  **‘Smokers and drinkers’**  **(n=16)** | **Cluster 3**  **‘Binge-drinkers and marijuana users’**  **(n=287)** | **Cluster 4**  **‘Smokers and binge-drinkers’**  **(n=57)** |
| --- | --- | --- | --- | --- |
|  | n (%) | n (%) | n (%) | n (%) |
| **Age******  *<=25*  *26+* | 360 (63)  317 (68) | 5 (1)  11 (2) | 180 (31)  107 (23) | 27 (5)  30 (7) |
| **Gender**  *Female*  *Male* | 467 (65)  199 (65) | 10 (1)  6 (2) | 200 (28)  85 (28) | 40 (6)  17 (5) |
| **Lonely******  *Never/rarely/sometimes*  *Frequently/always* | 550 (67)  127 (57) | 12 (2)  4 (2) | 212 (26)  75 (34) | 43 (5)  15 (7) |
| **Ethnicity**  *No*  *Yes* | 560 (66)  117 (61) | 14 (1)  2 (1) | 225 (27)  62 (32) | 48 (6)  10 (5) |
| **Region**  *Region of Coquimbo*  *Region of La Araucanía*  *Another region* | 147 (67)  437 (65)  71 (64) | 2 (1)  13 (2)  1 (1) | 61 (28)  184 (27)  34 (31) | 9 (4)  43 (6)  5 (4) |

***Note.*** **p value ≤ 0.05 one-way ANOVA. ±p value ≤ 0.05 Kruskal-Wallis. ** p value ≤ 0.05 Fishers Exact test.*

**Supplementary Table 4.** Bivariate analyses HRB cluster membership and covariates at T2.

|  | **Cluster 1**  **‘Lower risk’**  **(n=677)** | **Cluster 2**  **‘Smokers and drinkers’**  **(n=16)** | **Cluster 3**  **‘Binge-drinkers and marijuana users’**  **(n=287)** | **Cluster 4**  **‘Smokers and binge-drinkers’**  **(n=57)** |
| --- | --- | --- | --- | --- |
|  | n (%) | n (%) | n (%) | n (%) |
| **Age******  *<=25*  *26+* | 360 (63)  317 (68) | 5 (1)  11 (2) | 180 (31)  107 (23) | 27 (5)  30 (7) |
| **Gender**  *Female*  *Male* | 467 (65)  199 (65) | 10 (1)  6 (2) | 200 (28)  85 (28) | 40 (6)  17 (5) |
| **Lonely******  *Never/rarely/sometimes*  *Frequently/always* | 550 (67)  127 (57) | 12 (2)  4 (2) | 212 (26)  75 (34) | 43 (5)  15 (7) |
| **Ethnicity**  *No*  *Yes* | 560 (66)  117 (61) | 14 (2)  2 (1) | 225 (26)  62 (34) | 48 (6)  10 (5) |
| **Region**  *Region of Coquimbo*  *Region of La Araucanía*  *Another region* | 147 (67)  437 (65)  71 (64) | 2 (1)  13 (2)  1 (1) | 61 (28)  184 (27)  34 (31) | 9 (4)  43 (6)  5 (4) |
| **Depression** *Mean(SD)* | 7.04 (5.55) | 10.75 (5.91) | 8.03 (5.24) | 8.02 (6.67) |
| **Anxiety** *Mean(SD)*±* | 5.07 (5.23) | 4.75 (5.50) | 6.14 (5.15) | 6.62 (6.39) |
| **Stress** *Mean(SD)*±* | 7.91 (5.44) | 6 (5.48) | 9.23 (5.01) | 8.64 (6.70) |

***Note.*** **p value ≤ 0.05 one-way ANOVA. ±p value ≤ 0.05 Kruskal-Wallis. ** p value ≤ 0.05 Fishers Exact test.*

**Supplementary Table 5.** Multinomial logistic regression model estimating association between cluster membership at wave 0 and covariates, adjusting for classification error

| **Covariates** | **T0, *n* =1,038**  **Relative risk ratio (95% CI) ^a^** | | | |
| --- | --- | --- | --- | --- |
|  | **Cluster 1**  **‘Lower risk’**  ***n*= 677 (65.2%)** | **Cluster 2 ‘Drinkers and Smokers’**  ***n*= 16 (1.5%)** | **Cluster 3 ‘Marijuana users’**  ***n*= 287 (27.6%)** | **Cluster 4 ‘Smokers’**  ***n*= 58 (5.6%)** |
| **Age n (%)**  *<=25*  *26+* | *Ref cluster* | *Ref category*  2.92 (0.96 to 8.92) | *Ref category*  0.72 (0.54 to 0.97)*^b^ | *Ref category*  1.42 (0.81 to 2.49) |
| **Lonely n (%)**  *Never/rarely/sometimes*  *Frequently/always* | *Ref cluster* | *Ref category*  2.02 (0.61 to 6.71) | *Ref category*  1.40 (1.00 to 1.96)* | *Ref category*  1.72 (0.90 to 3.29) |

1. CI = confidence interval.
2. * = <0.05

**Supplementary Table 6.** Multinomial logistic regression model estimating association between cluster membership at T1 and covariates, adjusting for classification error

| **Covariates** | **T1, *n* =1,038**  **Relative risk ratio (95% CI) ^a^** | | | |
| --- | --- | --- | --- | --- |
|  | **Cluster 1**  **‘Lower risk’**  ***n*= 814 (78.4%)** | **Cluster 2 ‘Smokers and drinkers’**  ***n*= 13 (1.3%)** | **Cluster 3**  **‘Marijuana users’**  ***n*= 157 (15.1%)** | **Cluster 4 ‘Smokers’**  ***n*= 53 (5.1%)** |
| **Age n (%)**  *<=25*  *26+* | *Ref cluster* | *Ref category*  5.58 (1.29 to 24.14)*^b^ | *Ref category*  1.50 (1.01 to 2.22)* | *Ref category*  1.81 (0.94 to 3.47) |
| **Negative affect on economic or employment status n (%)**  *Not at all/a little/partly*  *A lot/entirely* | *Ref cluster* | *Ref category*  0.38 (0.05 to 3.17) | *Ref category*  1.30 (0.85 to 2.00) | *Ref category*  2.15 (1.12 to 4.15)* |
| **Depression** *Mean(SD)* | *Ref cluster* | 1.01 (0.85 to 1.21) | 1.01 (0.96 to 1.07) | 1.03 (0.95 to 1.13) |
| **Anxiety** *Mean(SD)* | *Ref cluster* | 1.31 (1.07 to 1.60)* | 1.00 (0.94 to 1.05) | 1.06 (0.96 to 1.17) |
| **Stress** *Mean(SD)* | *Ref cluster* | 0.81 (0.65 to 1.02) | 1.05 (0.99 to 1.11) | 0.97 (0.88 to 1.08) |

1. CI = confidence interval.
2. * = <0.05

**Supplementary Table 7.** Multinomial logistic regression model estimating association between cluster membership at T2 and covariates, adjusting for classification error

| **Covariates** | **T2, *n* = 430**  **Relative risk ratio (95% CI) ^a^** | | | |
| --- | --- | --- | --- | --- |
|  | **Cluster 1 ‘Lower risk’**  ***n*= 830 (80.0%)** | **Cluster 2 ‘Drinkers and Smokers’**  ***n*= 4 (0.01%)** | **Cluster 3**  **‘Marijuana users’**  ***n*= 144 (13.9%)** | **Cluster 4**  **‘Smokers’**  **n= 60 (5.8%)** |
| **Anxiety** Mean(SD) | *Ref cluster* | 1.05 (0.74 to 1.48) | 0.99 (0.91 to 1.09) | 1.07 (0.91 to 1.26) |
| **Stress** Mean(SD) | *Ref cluster* | 0.93 (0.68 to 1.29) | 1.07 (0.98 to 1.16) | 0.89 (0.77 to 1.04) |

1. CI = confidence interval.
2. * = <0.05
